# Dental Care Access Among Children and Adolescents With Autism Spectrum Disorder: A Cross Sectional Survey in Juiz de Fora, Brazil

**DOI:** 10.1111/scd.70158

**Published:** 2026-02-26

**Authors:** Pedro Mattos Cardoso, Laís Canêdo Martins, Fernanda Campos Machado, Camila Faria Carrada, Flávia Almeida Ribeiro Scalioni

**Affiliations:** ^1^ School of Dentistry Federal University of Juiz de Fora Juiz de Fora Minas Gerais Brazil; ^2^ Department of Social and Paediatric Dentistry, School of Dentistry Federal University of Juiz de Fora Juiz de Fora Minas Gerais Brazil; ^3^ Department of Paediatric Dentistry, School of Dentistry School of Medical Sciences – SUPREMA Juiz de Fora MG Brazil

**Keywords:** autism spectrum disorder, dental care for disabled, health services accessibility, pediatric dentistry, socioeconomic factors

## Abstract

**Aims:**

To investigate the conditions of access to dental care among children and adolescents with Autism Spectrum Disorder (ASD) in Juiz de Fora, Brazil, and to identify the main barriers faced by this population.

**Methods and Results:**

This cross sectional study used a convenience sampling strategy. Data were collected from 130 parents or caregivers of children and adolescents with ASD through a structured questionnaire disseminated via institutions and digital platforms. The instrument was developed based on previous studies and reviewed by experts to ensure content validity. Descriptive analyses and Pearson's chi‐square (*χ* ^2^) tests were performed. Most children with ASD (70.8%) had received dental care at least once, but 56.2% did not undergo regular follow‐up. The main barriers were difficulty finding specialized professionals (54.5%), financial constraints (42.0%), and behavioral limitations (23.9%). Younger children and those with higher family income, level 1 support, and verbal communication showed better adherence and behavior during dental care. Associations are reported without effect sizes or confidence intervals, as these analyses were not included in the study design.

**Conclusion:**

Although most children and adolescents with ASD in Juiz de Fora had received dental care, ongoing barriers hinder consistent access, as identified through associative—not causal—relationships, given the cross sectional nature of the study. The shortage of specialized professionals, behavioral challenges, and financial difficulties remain major barriers to access. Early age, verbal communication, and higher family income were associated with, rather than predictive of, better access and adherence.

## 1 Introduction

Autism Spectrum Disorder (ASD) is a neurodevelopmental condition characterized by persistent deficits in communication and social interaction, restricted and repetitive behaviors, and atypical responses to sensory stimuli [1, 2, 3, 4]. Although its clinical features are well described in the literature, the essential issue for oral health research lies not in the diagnostic presentation itself, but in how these characteristics influence access to dental services. Recent evidence highlights that individuals with ASD continue to face substantial challenges in obtaining timely and adequate oral health care, despite advances in awareness and diagnostic capacity [5, 6, 7, 8].

Children and adolescents with ASD often require extensive caregiver involvement for daily routines, including oral hygiene [6, 7]. These factors contribute to higher risk of oral conditions such as caries, periodontal disease, and parafunctional habits [7, 8, 9]. However, while the clinical implications of ASD for oral health are well documented, important gaps remain regarding access to dental services, particularly in underserved or regionally diverse populations. Recent systematic reviews have emphasized persistent disparities, especially related to socioeconomic status, lack of trained dental professionals, and organizational barriers in dental settings [10, 11, 12]. These recent studies were incorporated to strengthen the scientific background and update the evidence base supporting this research.

In Brazil, access to oral health care is mainly organized through the Unified Health System (Sistema Único de Saúde—SUS), which provides free dental services primarily at the primary care level, with referral to specialized care when needed. Alongside the public system, private dental services also play an important role, although access depends largely on out‐of‐pocket payment or private insurance, which may restrict utilization among socioeconomically vulnerable families. For individuals with ASD, this mixed public‐private model may intensify existing barriers to specialized dental care, as reported in international and Brazilian studies on access and service utilization [1, 3, 4, and 11]. Understanding this organizational context is essential for interpreting patterns of dental care access in the Brazilian setting.

Juiz de Fora, a midsized city with mixed public and private dental service provision, has no previous studies evaluating access to dental care for individuals with ASD. The decision to conduct the study in this municipality is therefore justified by the absence of epidemiological data, the regional distribution of specialized services, and the need to support local health planning.

In this context, the present study aimed to investigate access to dental care among children and adolescents with ASD in Juiz de Fora, to identify the main barriers perceived by caregivers, and to examine whether sociodemographic, socioeconomic, and ASD‐related characteristics were associated with patterns of access and behavior during dental appointments. In alignment with these objectives, the null hypothesis states that such characteristics would not be associated with access to dental care in this population.

## 2 Material and Methods

### 2.1 Ethical Considerations

The study was approved by the Human Research Ethics Committee of the Federal University of Juiz de Fora (CEP/UFJF), under CAAE 66908723.9.0000.5147 and opinion no. 5.889.332, issued on January 26, 2023. All procedures complied with the principles of the Declaration of Helsinki. Participation was voluntary, and electronic informed consent was obtained from all parents/caregivers prior to completion of the online questionnaire.

### 2.2 Study Design and Participants

This was a quantitative, descriptive, cross sectional study conducted between November 2024 and April 2025. The study rationale was incorporated, emphasizing the absence of local data on ASD‐related dental access in Juiz de Fora and the need for contextualized evidence to support regional health planning. The study population consisted of parents or caregivers of children and adolescents with a confirmed diagnosis of ASD aged 0–18 years, residing in Juiz de Fora, Minas Gerais, Brazil. Although the overall sample was recruited by convenience, participants were randomly selected within the identified population, and a minimum sample size of 100 participants was defined a priori to ensure adequate representation across key demographic factors, such as age, sex, and caregiver characteristics. Because convenience sampling was used, the generalizability of the findings may be limited.

### 2.3 Data Collection

Data were collected through a structured, self‐administered electronic questionnaire developed by the researchers based on previous studies. The instrument underwent content validation by two experts in pediatric dentistry and public health prior to distribution. The instrument consisted of two sections with a total of 38 multiple‐choice questions:

**Part I**: sociodemographic information and characteristics of the child/adolescent and their parents/caregivers;
**Part II**: access to and experiences with dental care services.

Initially, an electronic database was compiled with contact information (email addresses and telephone numbers, when available). The institutions contacted included both public (governmental) and private organizations that provide specialized services for individuals with ASD in Juiz de Fora. Emails were sent to the directors of these institutions, who were invited to share the study objectives and the survey link with eligible parents/caregivers. Additionally, the questionnaire link was disseminated through the researchers’ social media accounts (Instagram and Facebook) and distributed directly via WhatsApp. Participation required prior electronic informed consent, presented at the beginning of the form. Recruitment through institutions and social media may have introduced selection bias by favoring more engaged or resourceful caregivers. Moreover, all information was self‐reported and may therefore be subject to recall or social desirability bias. Access to dental care and regular follow‐up were recorded as binary outcomes, which may oversimplify the complexity of care experiences.

### 2.4 Inclusion and Exclusion Criteria

Inclusion criteria were parents or caregivers of children/adolescents aged 0–18 years, residing in Juiz de Fora, who were able to understand and respond to the questionnaire and who reported a previous formal diagnosis of ASD established by a qualified health professional. Exclusion criteria included parents/caregivers with potential conflicts of interest that could bias their responses, such as involvement in organizations related to dentistry or ASD.

### 2.5 Data Analysis

Data were entered into a database and analyzed using the Statistical Package for the Social Sciences (SPSS), version 21.0 for Windows (SPSS Inc., Chicago, IL, USA). All submitted questionnaires were complete and eligible for analysis; therefore, no losses due to incomplete responses were observed during the data collection process. Descriptive analysis was performed, with absolute and relative frequencies for categorical variables and mean and standard deviation for numerical variables. Missing data were handled using available‐case analysis. Analyses were performed using all available responses for each variable, and percentages were calculated based on the number of valid observations. Associations between sociodemographic, socioeconomic, and ASD‐related variables and access to dental care were tested using Pearson's chi‐square (*χ* ^2^) test. A significance level of *p* < 0.05 was adopted. Because the study had an exploratory design, analyses were restricted to descriptive statistics and Pearson's chi‐square (*χ* ^2^) tests. Multivariate models were not applied, which limits control for potential confounders such as age, income, and support level.

## 3 Results

Due to the use of multiple recruitment strategies, including institutional dissemination and social media, the total number of questionnaires distributed could not be determined, and therefore a response rate could not be calculated. A total of 130 caregivers of children and adolescents with ASD participated in the study. Most respondents were mothers (86.9%), predominantly non‐White (55.4%), with over nine years of education (86.2%) and from households supported by up to three members (56.2%). Additionally, Table 1 presents the characteristics of the children and adolescents with ASD.

**TABLE 1:** Personal data, sociodemographic characteristics, and ASD information (*N* = 130), Brazil, 2025.

	Distribution	
Variables	*N*	%
**Respondent's sex**		
Male	13	10.0
Female	117	90.0
**Respondent's age**		
Up to 40 years old	67	51.5
Over 40 years old	63	48.5
**Total monthly household income (considering the income of all residents)?**		
Less than 1 minimum wage (less than R$1100.00)	18	13.8
From 1 to 2 minimum wages (R$1100.00 to R$2200.00)	51	39.2
More than 2 to 3 minimum wages (R$2200.01 to R$3300.00)	15	11.6
More than 3 to 5 minimum wages (R$3300.01 to R$5500.00)	15	11.6
More than 5 minimum wages (more than R$5500.00)	31	23.8
**Sex of the child/adolescent with ASD**		
Male	100	76.9
Female	30	23.1
**Age of the child/adolescent with ASD**		
Up to 10 years old	85	65.4
Over 10 years old	45	34.6
**Ethnicity of the child/adolescent with ASD**		
White	72	55.4
Non‐white	58	44.6
**Is the child/adolescent with ASD currently enrolled in an educational institution?**		
Yes	122	93.8
No	8	06.2
**If yes, is the institution public or private?**		
Public	92	75.4
Private	30	24.6
**At what age was the child/adolescent diagnosed with ASD?**		
Mean (Standard deviation)	4,27 (±3,082)	
**What level of support was the child/adolescent diagnosed with?**		
Level 1	56	43.1
Level 2	43	33.1
Level 3	27	20.8
Other	4	3.0
**Does the child/adolescent with ASD have any other psychiatric comorbidity?**		
Yes	73	56.2
No	57	43.8
**Is the child/adolescent currently receiving specialized medical care for ASD?**		
Yes, with a psychiatrist	27	20.8
Yes, with a neurologist	92	70.8
Yes, with another specialty	5	03.8
No	6	04.6
**Is the child/adolescent currently receiving speech therapy?**		
Yes	70	53.9
No	58	44.6
Do not know	2	01.5
**Is the child/adolescent currently receiving physical therapy?**		
Yes	52	40.0
No	74	56.9
Do not know	4	03.1
**Is the child/adolescent currently receiving occupational therapy?**		
Yes	60	46.20
No	68	52.30
Do not know	2	01.5
**Is the child/adolescent currently receiving psychological therapy?**		
Yes	91	70.0
No	38	29.2
Do not know	1	00.8
**Does the child/adolescent with ASD present difficulties in the following areas (check all that apply)?**		
Attention and concentration	99	76.1
Learning	66	50.7
Time management	54	41.5
Task organization	75	57.6
Communication	77	59.2
Eating	61	46.9
Sleep	37	28.4
Irritability	66	50.7
Socialization	73	56.1
None of these difficulties	1	0.7
Other	5	3.8
Prefer not to answer	0	00.0
**Does the child/adolescent with ASD present strengths or high abilities in the following areas (check all that apply)?**		
Attention and concentration	15	11.5
Learning	50	38.4
Time management	7	5.3
Task organization	17	13.0
Communication	25	19.2
Eating	24	18.4
Sleep	24	18.4
Irritability	5	3.8
Socialization	20	15.3
None of these difficulties	8	6.1
Other	26	20.0
Prefer not to answer	11	8.4
**Is the child/adolescent with ASD verbal or nonverbal?**		
Verbal	90	69.2
Nonverbal	40	30.8

*Notes*: Due to missing data, analyses were conducted using available‐case analysis. Percentages are calculated based on the number of valid responses for each variable.

*N*: absolute number; %: frequency.

Psychiatric comorbidities were reported by caregivers and included attention‐deficit/hyperactivity disorder (ADHD), anxiety disorders, intellectual disability, epilepsy, and other previously diagnosed conditions.

Regarding dental care, 70.8% had attended at least one dental appointment, most commonly in private clinics. However, more than half (56.9%) did not maintain regular follow‐up. The main barriers reported were difficulty finding specialized professionals (66.6%), financial difficulties (51.3%), and limitations related to the child or adolescent (29.1%). Only about one‐quarter of dentists providing care were trained to treat this population (Table 2).

**TABLE 2:** Data regarding dental care (*N* = 130), Brazil, 2025.

	Distribution	
Variables	*N*	%
**Has the child/adolescent with ASD ever received dental care from a dentist at least once in their lifetime?**		
Yes	92	70.8
No	38	29.2
**If not, what factors have prevented this dental care?**		
Difficulty in finding a specialized professional	35	92.1
Distance between the dental office and the residence	2	5.2
Lack of time or a person available to accompany the child/adolescent with ASD	4	10.5
Limitations of the child/adolescent	15	39.4
Fear of dental treatment	20	52.6
Financial difficulties	29	76.3
Lack of perceived need	3	7.8
Other	13	34.1
**If yes, in what type of dental office does the child/adolescent with ASD receive care?**		
Private practice	54	58.7
Public practice	38	41.3
**Does the child/adolescent with ASD attend regular dental check‐ups?**		
Yes	56	43.1
No	74	56.9
**If not, what are the main barriers to maintaining regular follow‐up?**		
Difficulty in finding a specialized professional	48	66.6
Distance between the dental office and the residence	6	8.3
Lack of time or a person available to accompany the child/adolescent with ASD	7	9.7
Limitations of the child/adolescent	21	29.1
Fear of dental treatment	19	26.3
Financial difficulties	37	51.3
Lack of perceived need	4	5.5
Other	17	23.6
**Is the dentist who provides care specialized in treating children/adolescents with ASD?**		
Yes	31	23.9
No	56	43.1
Do not know	43	33.0
**Has the child/adolescent with ASD ever required conscious sedation or general anesthesia during dental treatment?**		
Yes	28	23.9
No	82	70.1
Do not know	20	16.0
**How do you evaluate the dental care provided by the dentist?**		
Very good/good	78	60.0
Fair	13	10.0
Poor/very poor	2	1.5
Prefer not to answer	14	10.8
Missing data	23	17.7
**How do you evaluate the behavior of the child/adolescent with ASD in the dentist's waiting room?**		
Very good/good	47	36.2
Fair	39	30.0
Poor/very poor	14	10.8
Prefer not to answer	11	8.5
Missing data	19	14.6
**How do you evaluate the behavior of the child/adolescent with ASD in the dentist's office?**		
Very good/good	52	40.0
Fair	38	29.2
Poor/very poor	11	08.5
Prefer not to answer	13	10
Missing data	16	12.3
**How do you evaluate the behavior of the child/adolescent with ASD during the dental appointment?**		
Very good/good	56	43.1
Fair	30	23.1
Poor/very poor	15	11.5
Prefer not to answer	14	10.8
Missing data	15	11.5
**What is the most challenging factor for dental care?**		
Difficulty in finding a specialized professional	42	35.9
Distance between the dental office and the residence	1	0.9
Lack of time or a person available to accompany the child/adolescent with ASD	5	04.3
Limitations of the child/adolescent	17	14.5
Fear of dental treatment	11	09.4
Financial difficulties	31	26.5
Lack of perceived need	0	00.0
Other	10	08.6

*Note*: Due to missing data, analyses were conducted using available‐case analysis. Percentages are calculated based on the number of valid responses for each variable.

*N*: absolute number; %: frequency.

Significant associations were observed for age and family income. Younger children (≤10 years) were more likely to have received dental care (59.8% vs. 40.2%, *p* = 0.043) and to attend regular follow‐up (51.8% vs. 48.2%, *p* = 0.005). Children from higher‐income households (>2 minimum wages) were also more likely to receive dental care (54.3% vs. 45.7%, *p* = 0.012) and maintain follow‐up (60.7% vs. 39.3%, *p* = 0.012) (Table 3).

**TABLE 3:** Factors associated with demographic, socioeconomic, and ASD characteristics and dental care access/continuity (*N* = 130), Brazil, 2025.

	Ever received dental care			Receives regular dental follow‐up		
	Yes (%)	No (%)	CI 95% *p*‐value	Yes (%)	No (%)	CI 95% *p*‐value
**Child/adolescent's sex**						
Female	22 (23.9)	8 (21.1)	0.72–1.54	13 (23.2)	15 (20.8)	0.70–1.55
Male	70 (76.1)	30 (78.9)	0.821	43 (76.8)	57 (79.2)	0.830
**Child/adolescent's age**						
Up to 10 years old	55 (59.8)	0 (78.9)	1.02–2.73	9 (51.8)	55 (76.4)	1.29–4.22
Over 10 years old	37 (40.2)	8 (21.1)	0.043^a^	27 (48.2)	17 (23.6)	0.005^a^
**Ethnicity**						
White	53 (58.2)	19 (50.0)	0.98–2.05	37 (66.1)	35 (49.3)	0.97–1.98
Non‐white	38 (41.8)	19 (50.0)	0.439	19 (33.9)	36 (50.7)	0.072
**Family income**						
Up to 2 minimum wages	42 (45.7)	27 (71.1)	1.18–3.74	22 (39.3)	45 (62.5)	1.16–3.38
More than 2 minimum wages	50 (54.3)	11 (28.9)	0.012^a^	34 (60.7)	27 (37.5)	0.012^a^
**Support level**						
Level 1	44 (49.4)	12 (32.4)	0.78–1.79	27 (49.1)	28 (40.6)	0.60–1.12
Level 2	29 (32.6)	14 (37.8)	0.060	19 (34.5)	24 (34.8)	0.232
Level 3	16 (18.0)	11 (29.7)		9 (16.4)	17 (24.6	
**Verbal communication**						
Yes	68 (73.9)	22 (57.9)	0.98–2.05	42 (75.0)	48 (66.7)	0.82–1.78
No	24 (26.1)	16 (42.1)	0.095	14 (25.0)	24 (33.3)	0.335

^a^ *p* < 0.05 Pearson's Chi‐square (*χ* ^2^) test.

Behavioral outcomes during dental visits were strongly influenced by ASD characteristics. Behavior in the waiting room, dental office, and during dental appointments was assessed based on caregiver report, using subjective categories provided in the questionnaire, without the application of standardized behavioral assessment instruments. Children with verbal communication and those requiring level 1 support were significantly more often rated as having very good/good behavior across the waiting room, dental office, and appointments (all *p* < 0.05). In contrast, nonverbal children and those requiring level 3 support were more frequently assessed as poor/very poor (Table 4).

**TABLE 4:** Factors associated with ASD characteristics and parents’/caregivers’ perception of dental care (*N* = 130), Brazil, 2025.

	Dentist's performance evaluation				CI 95% *p*‐value	Child/adolescent behavior in the waiting room				CI 95% *p*‐value	Child/adolescent behavior in the dental office				CI 95% *p*‐value	Child/adolescent behavior during appointment				*p*‐value
	Very good/good (%)	Fair (%)	Poor/very poor (%)	Prefer not to answer (%)		Very good/good (%)	Fair (%)	Poor/very poor (%)	Prefer not to answer (%)		Very good/good (%)	Fair (%)	Poor/very poor (%)	Prefer not to answer (%)		Very good/good (%)	Fair (%)	Poor/very poor (%)	Prefer not to answer (%)	
**Support**																				
Level 1	35 (46.7)	8 (61.5)	1 (50.0)	4 (30.8)	0.91–1.81	27 (58.7)	17 (47.2)	1 (7.1)	5 (45.5)	1.55–5.24	32 (64.0)	12 (33.3)	1 (9.1)	6 (46.2)	1.33–3.99	32 (59.3)	10 (35.7)	2 (13.3)	6 (42.9)	1.32–3.88
Level 2	27 (36.0)	3 (23.1)	0 (0.0)	4 (30.8)		15 (32.6)	14 (38.9)	3 (21.4)	2 (18.2)		14 (28.0)	14 (38.9)	5 (45.5)	3 (23.1)		18 (33.3)	11 (39.3)	4 (26.7)	4 (28.6)	
Level 3	13 (17.3)	2 (15.4)	1 (50.0)	5 (38.5)	0.148	4 (8.7)	5 (13.9)	10 (71.4)	4 (36.4)	0.001^a^	4 (8.0)	10 (27.8)	5 (45.5)	4 (30.8)	0.003^a^	4 (7.4)	7 (25.0)	9 (60.0)	4 (28.6)	0.003
**Verbal**																				
Yes	57 (73.1)	10 (76.9)	1 (50.0)	5 (35.7)	1.03–2.55	37 (78.7)	31 (79.5)	4 (28.6)	6 (54.5)	1.98–5.91	40 (76.9)	29 (76.3)	4 (36.4)	7 (53.8)	1.07–2.73	45 (80.4)	24 (80.0)	4 (26.7)	7 (50.0)	1.06–2.70
No	21 (26.9)	3 (23.1)	1 (50.0)	9 (64.3)	0.038^a^	10 (21.3)	8 (20.5)	10 (71.4)	5 (45.5)	<0.001^a^	12 (23.1)	9 (23.7)	7 (63.6)	6 (46.2)	0.024^a^	11 (19.6)	6 (20.0)	11 (73.3)	7 (50.0)	0.024

^a^ *p* < 0.05 Pearson's Chi‐square (*χ* ^2^) test. CI: Confidence interval.

Overall, caregivers expressed relatively high satisfaction: 60.0% rated dental care as very good/good, particularly when services were accessible and provided by professionals prepared to manage children and adolescents with ASD.

## 4 Discussion

Access to dental care for children and adolescents with ASD remains a multifactorial challenge influenced by individual characteristics, family resources, and the organization of health services. Rather than reiterating descriptive findings, this discussion prioritizes critical interpretation of the observed associations in light of existing evidence and contextual constraints. Previous studies have consistently shown that access to dental services for individuals with ASD is shaped by the interaction between socioeconomic conditions, behavioral characteristics, and service organization [12, 13, 14, 15].

Socioeconomic conditions were associated with patterns of dental care access, corroborating earlier studies that identified income and parental education as relevant factors influencing service utilization among children with ASD [11, 12, 13, 14]. However, contradictory findings have been reported in contexts with more structured public oral health systems, where socioeconomic disparities appear attenuated [16]. This contrast suggests that the influence of income on access is context‐dependent and mediated by health system organization rather than individual capacity alone.

Behavioral and communication characteristics were associated with caregiver‐reported experiences during dental appointments, findings that align with literature describing sensory sensitivities, communication difficulties, and behavioral challenges as barriers to oral health care [9, 10, 11]. These associations should be interpreted cautiously, as they are based on caregiver perceptions rather than objective clinical assessments. While several studies describe similar difficulties, others demonstrate that professional training, behavioral management strategies, and adapted dental environments can substantially improve dental care experiences for individuals with ASD [17, 18]. The coexistence of convergent and divergent findings highlights the need to avoid deterministic interpretations of ASD‐related behaviors.

Age‐related factors also emerged as relevant for interpreting access to dental care. In the present study, most children were aged 10 years or younger, and the mean age at diagnosis was relatively early when compared with settings characterized by delayed recognition. Although this finding may be considered favorable, international guidelines continue to recommend ASD diagnosis by the age of 3, indicating that opportunities for even earlier identification and intervention remain [15, 19].

These results are consistent with Corridore et al. [5] and Sami et al., [15], who reported greater complexity of dental care among children with higher levels of functional impairment. In addition, younger children (≤10 years) were more likely to have accessed dental care and to maintain follow‐up, suggesting stronger parental engagement during early childhood. However, adherence appeared to decline with increasing age, possibly reflecting cumulative behavioral challenges, heightened sensory sensitivities, and increasing caregiver burden over time, as described in previous studies [4, 20]. This pattern underscores that early access, although important, does not guarantee continuity of care throughout adolescence.

Satisfaction with oral health services should be interpreted with caution, as it may reflect response bias related to access rather than an objective assessment of service quality. In settings where barriers to dental care are common, caregivers who succeed in obtaining services may report higher satisfaction levels primarily due to gratitude for access itself. Similar patterns have been described in studies examining caregiver perceptions and access to oral health care within public health systems [3, 4, and 16].

The predominance of low‐income caregivers in the present sample suggests that the public health sector constitutes the primary pathway for access to oral health services for individuals with ASD. This pattern aligns with the organization of the Brazilian Unified Health System, in which dental care is predominantly delivered through public primary health care services, with referral to specialized centers when needed. Consistent findings have been reported in Brazilian and international studies, indicating that families with lower socioeconomic status rely more heavily on public dental services for children with ASD [11, 16].

This pattern contrasts with findings from settings in which access to dental care for individuals with ASD is primarily mediated by private insurance coverage or out‐of‐pocket payment. In these contexts, utilization of dental services is more closely associated with family income and insurance status, which may intensify inequalities in access [3, 4, and 10]. These contrasts highlight the role of health system organization and socioeconomic conditions in shaping access to oral health care for individuals with ASD.

Methodological characteristics of the present study must be carefully considered when interpreting these findings. The use of convenience sampling, as reported in similar caregiver‐based studies [12, 13, 14, 15], may have favored families more engaged with health services or support networks, potentially overestimating access and continuity of care. Recruitment through social media and specialized institutions may have excluded caregivers with limited digital access or weaker institutional ties, introducing selection bias. Additionally, reliance on self‐reported data, common in access‐to‐care research [9, 10, 11], introduces recall and social desirability bias, which may have influenced reports of access, follow‐up, and behavior.

The cross sectional design represents another important limitation. Although associations between socioeconomic variables, behavioral characteristics, and access to dental care were identified, these findings do not imply causal relationships and should not be interpreted as explanatory. Similar methodological constraints have been acknowledged in previous cross sectional investigations on oral health access among individuals with ASD [11, 12, 13, 14]. All causal language was intentionally removed in this revised discussion to ensure consistency with the study design. Longitudinal and mixed‐methods approaches are therefore needed to clarify temporal relationships and underlying mechanisms influencing access and continuity of dental care [19, 21].

From a clinical and policy perspective, the findings support specific and actionable recommendations rather than general statements. These include structured training programs for dental professionals focusing on ASD‐related behavioral management, development of referral pathways linking primary care to specialized services, and sensory‐adapted dental environments to reduce distress during appointments [17, 18]. At the policy level, strengthening collaboration between oral health services, educational institutions, and caregiver support networks may reduce access barriers, particularly in midsized urban settings [20, 22].

Despite its contributions, this study has important limitations. The absence of a control group limits comparative interpretation, and the lack of objective clinical oral health measures prevents direct assessment of oral health status in relation to reported access to care. These limitations, frequently observed in studies addressing access to dental services among individuals with special health care needs [10, 11, 12], reduce external validity and preclude causal inference. Therefore, the findings should be interpreted as exploratory, highlighting the need for future studies incorporating standardized clinical assessments, comparative designs, and broader sampling strategies to strengthen evidence on access to dental care for individuals with ASD [23].

## 5 Conclusion

This study provides ASD in Juiz de Fora, highlighting associations between sociodemographic, socioeconomic, and ASD‐related characteristics and reported access and follow‐up. Given the methodological constraints, the findings should be interpreted cautiously and cannot be used to infer causal relationships. Despite these limitations, the results underscore persistent barriers related to service availability, behavioral challenges, and socioeconomic context. Future research should prioritize longitudinal designs and intervention‐based studies to evaluate strategies aimed at improving access, continuity of care, and oral health outcomes for individuals with ASD.

## Author Contributions

Pedro Mattos Cardoso and Flávia Almeida Ribeiro Scalioni conceived the study design. Pedro Mattos Cardoso, Laís Canêdo Martins, and Flávia Almeida Ribeiro Scalioni collected, analyzed, and interpreted the data and drafted the manuscript. Fernanda Campos Machado and Camila Faria Carrada provided critical revisions and intellectual input. All authors read and approved the final version of the manuscript.

## Funding

This research received no external funding. All authors are affiliated with the Federal University of Juiz de Fora (UFJF), which provided only institutional support.

## Conflicts of Interest

The authors declare no conflict interests.

## Data Availability

The datasets generated and analyzed during the current study are available from the corresponding author on reasonable request.
